# Ocular Manifestations and Potential Treatments of Alport Syndrome: A Systematic Review

**DOI:** 10.1155/2022/9250367

**Published:** 2022-09-08

**Authors:** Rahul Ramakrishnan, Atira Shenoy, Damon Meyer

**Affiliations:** ^1^Nova Southeastern University Dr. Kiran C. Patel College of Allopathic Medicine, Fort Lauderdale, FL, USA; ^2^Nova Southeastern University Dr. Kiran C. Patel College of Osteopathic Medicine, Fort Lauderdale, FL, USA; ^3^California Northstate University, College of Health Sciences, 2910 Prospect Park Dr., Rancho Cordova, CA 95670, USA

## Abstract

**Objectives:**

Alport syndrome (AS) is a severe, rare hereditary disorder that can lead to end-stage renal disease, auditory degeneration, and ocular abnormalities. Despite extensive research on AS in relation to auditory and renal disorders, more research is needed on the ocular presentations of AS. This systematic review aims to summarize the common ocular abnormalities in patients with AS and to explore the potential treatment options for these irregularities.

**Methods:**

The PubMed, MEDLINE, and EMBASE databases were systematically searched from January 1977 to April 2022. Only papers that were published in the English language and explored the ocular abnormalities in AS patients were selected. We manually searched reference lists of included papers for additional studies.

**Results:**

A total of 23 articles involving 195 patients were included in this review. The common ocular manifestations in AS patients are lenticonus, macular holes, fleck retinopathy, and thinning of the macula. Although published literature has described the use of cataract surgeries and vitrectomies as standard surgical techniques to alleviate ocular abnormalities in non-AS patients, it must be noted that surgical techniques have not been evaluated in a large research study as a solution for AS abnormalities. Another prospective treatment for AS is gene therapy through the reversion of causative *COL4* variants to wild type or exon-skipping therapy for *X*-linked AS with *COL4A5* truncating mutations. Gene therapy, however, remains unable to treat alterations that occur in the fetal and early development phase of the disease.

**Conclusions:**

The review found no definitive conclusions regarding the efficacy and safety of surgical techniques and gene therapy in AS patients. Recognition of ocular abnormalities through an ophthalmic examination with an optical coherence tomography (OCT) and slit-lamp examination is critical to the medical field, as ophthalmologists can aid nephrologists and other physicians in diagnosing AS. Early diagnosis and care can minimize the risk of detrimental ocular outcomes, such as blindness and retinal detachment.

## 1. Background

Alport syndrome (AS) is a rare (1 : 5,000) heterogeneous genetic disorder characterized by kidney failure, hearing loss, and eye abnormalities [1]. AS is inherited and mainly induced by a mutation within one of the collagen genes; *COL4A3* and *COL4A4* are located on chromosome 2 while *COL4A5* is located on the *X*-chromosome [2]. The *X*-linked form of the disease is the most common, representing nearly 80% of all AS cases, while the autosomal forms of the disease, which can be dominant or recessive depending on the gene variant, account for the remaining 20% [2, 3]. Moreover, while males are typically more severely affected, females who are heterozygous for *COL4A5* variants will still present symptoms that are typically milder and show a slower progression compared to males [2, 3]. The *COL4A3*, *COL4A4*, and *COL4A5* genes encode collagen IV *α*3, *α*4, and *α*5 chains, respectively [2]. The collagen IV *α*345 chains form a network that serves as a prominent structural component in the glomerular basement membranes of the eyes, ears, and kidneys. Sequence variants in the *COL4A5* gene of AS patients have been curated by the University of Utah and currently include 807 entries [4]. Approximately 94.4% of these variants are considered pathogenic and include nucleotide insertions, deletions, splice site alterations, and amino acid changes leading to nonsense and missense changes that impact the amount of functional type IV collagen [4]. Variants in *COL4A3* and *COL4A4* genes are curated on the Leiden Open Variation Database (LOVD) with 257/579 of *COL4A3* and 222/558 of *COL4A4* considered to be likely or confirmed pathogenic and show a similar distribution of nucleotide and amino acid changes observed in *COL4A5* [5]. Studies have reported little to no discernible phenotypes in individuals possessing recessive digenic heterozygous mutations in both *COL4A3* and *COL4A4* [6, 7]. Interestingly, a pathogenic *COL4A5* variant combined with a recessive heterozygous pathogenic variant in *COL4A3* or *COL4A4* is associated with proteinuria that is more severe when compared to individuals possessing only the *COL4A5* variant [8]. This suggests that the variation in phenotypes observed in AS patients can, in part, be attributed to the amount of remaining functional type IV collagen chains in affected tissues.

The ocular symptoms AS patients present include lenticonus, macular flecks, retinopathy, corneal changes, macular thinning, macular holes, retinal thinning, cataract, anterior lens capsule changes, and dull macular reflex [9–12]. Lenticonus is defined as a protrusion of the anterior or posterior lens surface that can be in one eye (unilateral) or both eyes (bilateral). AS patients will usually present with a bilateral anterior lenticonus that may have perimacular retinal flecks but in rare circumstances can have simultaneous anterior and posterior lenticonus [9, 10]. Retinopathies commonly observed in AS include central fleck and peripheral fleck retinopathies that have little to no effect on visual acuity and do not require treatment, while corneal changes such as recurrent corneal erosions and posterior polymorphous corneal dystrophy are less common but can impair vision [11]. Macular thinning and retinal thinning are primarily image-based findings that show the central retina (macula) or the peripheral retina becoming thin and do not affect vision, while macular holes are defined as retinal defects in the central fovea are uncommon in AS patients and do not respond well to surgery, which can result in permanent vision loss [11–16]. Anterior lens capsule (ALC) abnormalities include the dehiscence, rupture, or thinning of the ALC that protects the lens [12, 17]. Finally, cataracts occur following the healing of small partial splits that rupture from the lenticonus, and dull macular reflex is the loss of the proper foveal reflex and has been associated with early-onset renal failure [18, 19].

Due to its rarity, there is no standard, effective treatment for AS. AS is generally diagnosed during childhood due to hematuria and proteinuria and not due to ophthalmologic reasons. Since the condition is genetic, many prior researchers have attempted to investigate whether gene therapy would be an avenue for treatment [20]. However, it must be noted that gene therapy would not treat the alterations that occur during the fetal and early developmental phase of the disease. In addition, gene therapy is still in the early phases of clinical utility for the treatment of certain genetic diseases. Regardless, gene therapy research and its potential implementation into medicine are showing promising results and could be used to treat and manage genetic diseases in the future.

The majority of prior reports on AS have focused on regions other than the eye, such as the kidney and ears. However, since collagen is a crucial protein in the eyes due to its necessity in the clear cornea, sclera, and wound healing, the *COL4A3-5* mutations in AS patients would predictably result in vision deterioration [21]. A few reports have analyzed large cohorts of patients with *X*-linked AS and reported on the specific ocular complications of lenticonus, maculopathy, cataracts, and ocular lesions [22, 23]. Therefore, this systematic review will focus on ocular abnormalities in AS and provide a summary of what is currently known, which includes an examination of the medical management and potential treatment options referenced by prior research papers.

## 2. Methods

This systematic review was conducted in accordance with Preferred Reporting Items for Systematic Reviews and Meta-Analyses (PRISMA) guidelines [24, 25]. The protocol for the review was not registered.

### 2.1. Search Strategy and Selection Criteria

The cited articles were identified through the PubMed, MEDLINE, and EMBASE electronic databases from January 1977 until April 2022 using the following keywords: “Alport syndrome” and “macula” or “retinal thinning” or “lenticonus” or “retinopathy” or “ocular.” We also conducted manual searches of the reference lists of relevant papers to retrieve additional publications.

Identified papers were thoroughly screened by one author (RR) using the titles, abstracts, and inclusion and exclusion criteria mentioned below. The papers were independently reviewed by the second author (AS) to verify inclusion in the systematic review. Uncertainty regarding specific papers and whether they should be included in the review were resolved through discussions among all authors. When needed, complete manuscripts were obtained for additional analysis and decision-making.

**Figure 1:**
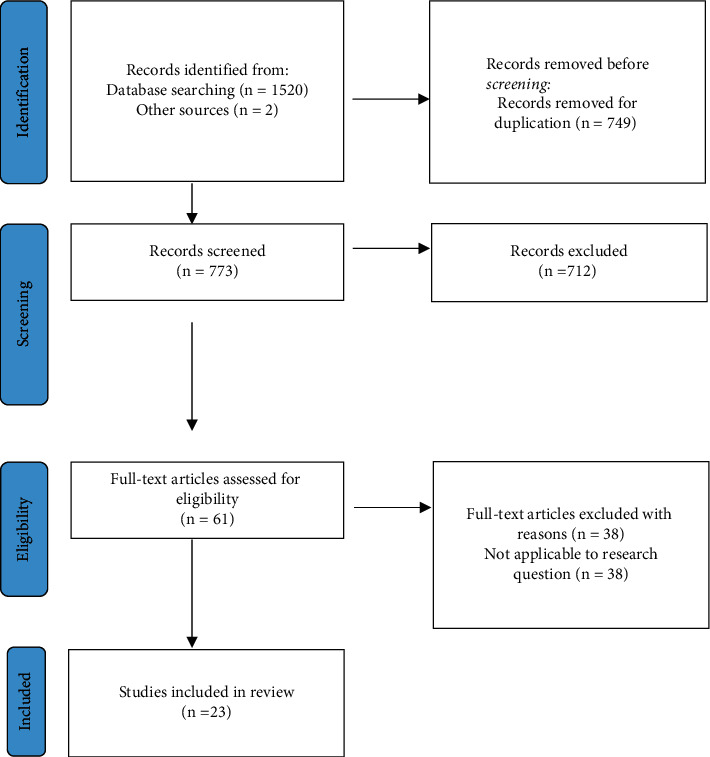
PRISMA flow diagram of the study selection process.

The inclusion criteria consisted of research papers and case study reports that related ocular abnormalities to Alport syndrome. In addition, only English language studies were included in the review. Although there was an English language restriction enforced, there was no demographic restriction. The exclusion criteria were as follows: grey literature such as abstracts from conferences and papers that mentioned Alport syndrome but did not mention ocular symptoms. For example, papers solely focused on renal failure and deafness symptoms were excluded. In addition, papers that mentioned complications other than ocular complications were carefully examined to ensure they met the extent of what was agreed upon by the authors. See Figure 1.

### 2.2. Data Extraction

A single reviewer collected and compiled data from the articles using Microsoft Excel and a developed data extraction sheet. Studies were analyzed to retrieve data regarding the first author, aims, methods, participants, limitations, ocular abnormalities, potential medical treatments, and characteristics of the study design. The extracted information was independently reviewed by the second reviewer. Any ambiguity was resolved through discussions among all authors of this paper. The outcomes analyzed would include the types of ocular abnormalities arising from AS, the rate of common and rare ocular abnormalities in AS patients, and the potential medical treatments.

### 2.3. Quality Assessment

Two independent reviewers used the Critical Appraisal Skills Programme (CASP) to assess the quality of evidence [26]. Any disagreements were resolved among all the authors. Papers with a low risk of bias were included. The Cochrane risk-of-bias tool for randomized trials was not utilized as there were no randomized controlled trials (RCTs) among the papers considered in this systematic review [27].

### 2.4. Data Synthesis and Analysis

Using the extracted information from Table 1 as well as the complete manuscripts of included studies, we were able to calculate the percentages of common and rare ocular abnormalities in AS patients who presented with ocular irregularities. These calculations allow accessibility and ease of understanding for patients and researchers with regard to the various complications that can present in AS patients. A meta-analysis was not undertaken due to outcome measures and the design of studies.

### 2.5. Patients and Public Involvement

No patients or members of the public were involved in this review.

## 3. Results

### 3.1. Study Selection and Study Characteristics

There was a total of 1522 papers initially identified following searches of the three electronic databases utilizing the aforementioned keywords. Following the removal of 749 duplicates, 773 papers were screened. During the screening process, 712 papers were excluded as they did not meet the inclusion criteria. When assessing the eligibility of the remaining 61 full-text articles, 38 studies were excluded as they were not applicable to the research question. Therefore, 23 studies were included in the review. This entire process is summarized as a flow diagram in Figure 1. Of the 23 included studies, twelve were case studies on an individual or a group of patients [30, 33, 35, 37, 39, 41, 43–47, 50], two were prospective studies[29, 38], three were retrospective studies [31, 32, 49], and six were mixed methodology studies that did not meet the previous three categories [28, 34, 36, 40, 42, 48] (Table 1). 20 studies reported AS ocular abnormalities only [28, 30–38, 40–49], and the remaining 3 studies reported both ocular abnormalities and potential medical treatments [29, 39, 50]. We did not identify any studies that confirmed a high success, standardized medical treatment for AS ocular abnormalities. None of the articles that were reviewed established a relationship between the severity of the nephritis or hearing loss and the ocular abnormalities mentioned. However, one paper suggested that commonly reported ocular manifestations such as lenticonus could be linked to nephropathy prognosis in AS patients [49].

### 3.2. Common Ocular Manifestations

Thirteen studies reported common ocular abnormalities in AS patients. Adıyeke et al. [28] conducted a study in the United States with 21 AS patients from ages 9 to 18 and found the commonly reported findings in all the patients to be lenticonus, retinopathy, cataracts, macular holes, and macular thinning. Gelisken et al. [34] investigated 13 AS patients and discovered that 12 of the patients had flecked retinopathy while 2 patients presented with retinal vascular tortuosity. Ahmed et al. [29] performed a similar study as Adıyeke's research group in Turkey with 32 AS patients and discovered similar abnormalities with the addition of retinal thinning. Fukukita et al. [32] investigated retinal changes in 26 AS patients living in Japan and discovered the same symptoms with the exclusion of retinal thinning and cataracts. Finally, several other studies by Gupta et al. [37] who studied 2 AS patients in India and Wong et al. who examined 1 AS patient in Australia [48] corroborated the lenticonus and retinopathies' presence. In total, these six studies showed 95 AS patients from six different countries with similar symptoms. Since the studies consisted of a diverse sample of patients, the findings are sufficient in providing the typical ocular manifestations observed in AS.

While the above research focused on lenticonus, retinopathies, and other common ocular abnormalities in AS, Gehrs et al. [33] and Karki and Shrestha [41] reported on 2 separate patients who had macular flecks (also known as retinal flecks), tiny lesions on the macula of the retina. Jeffrey et al. [40] also discovered flecks in 20 patients after conducting an electrophysical study. These studies demonstrate that retinal flecks are consistently found in patients with AS. Macular flecks are significant as Gehrs et al. and Karki et al. both mention how flecks can help diagnose retinopathies. However, macular flecks in AS patients are not directly associated with retinal dysfunction and do not require treatment. However, additional research on AS patients will be needed to determine the relationship between macular flecks and retinopathies that may require treatment. Hess et al. [38] examined the foveal pit and foveal avascular zone (FAZ) phenotypes in a study of 14 AS patients in a hospital in Germany. This report showed that AS presents with foveal hypoplasia with no FAZ, a representation of an underdeveloped macula, and an enlarged FAZ with staircase foveopathy. Hess et al. suggest that collagen IV, which is normally defective in AS patients, must be involved in foveal development and maturation.

Several studies have tried to link ocular abnormalities with AS to aid diagnosis. Fawzi et al. [31] described the ophthalmic manifestations in 9 AS patients, focusing on retinal findings such as vitelliform maculopathy, retinal flecks, and macular lesions. The paper concluded that AS diagnosis can be made through clinical examinations, particularly with findings of peripheral retinal flecks. Common ocular examinations that would lead to diagnosis would include optical coherence tomography (OCT) and fundus autofluorescence (FAF). OCT would provide a cross-sectional perspective of the retina to diagnose macular holes and other ocular abnormalities, while a FAF is necessary to analyze the structure and health of the retina's center and periphery. Mete et al. [42] studied 8 AS patients that presented with complications of lenticonus, retinal flecks, and macular holes. The report further confirmed the prevalence of ocular complications and the importance of clinical examinations in AS diagnosis. Xu et al. [49] described 13 AS patients with findings of lenticonus, macular flecks, cataracts, and nystagmus. While the paper originally investigated 32 AS patients, only 13 of the patients (40.6% of the original patients) presented with ocular manifestations. The authors elaborated that these complications can aid in the diagnosis of AS since common ocular manifestations such as lenticonus were also accompanied by complications in the renal and auditory systems. Finally, Govan [36] details the findings of 16 AS patients in the United Kingdom who were being treated for renal disease. The report found patients presenting with lenticonus and macular flecks. During ocular examinations, these complications presented as weakened macular reflexes. They also described how the diagnosis of AS can be made based on the presence of at least one of the following ocular features: anterior lenticonus, macular flecks in the internal limiting membrane, or peripheral coalescing flecks. This supports the conclusions by Fawzi et al. and Mete et al. that ocular examination can help in the diagnosis of AS but are careful to point out that the absence of the above ocular features does not exclude the diagnosis.

### 3.3. Rare Ocular Manifestations

Early reports of AS patients reported a rare presentation of abnormal macular pigmentation (AMP), a sign of macular degeneration which can progress into vision loss. Nielsen [44] investigated six AS patients from five different families in Denmark and reported that four of the six patients presented with AMP. Out of the four patients who presented with AMP, two of the patients were biologically related. In a separate study, Singh et al. [45] examined four AS patients from the same family in Southern India. The report from Singh et al. notes that the four patients had AMP. Singh et al. suggested an autosomal dominant pattern of inheritance with incomplete penetrance as the four Indian family members had the same rare AMP condition. No treatments were proposed in either study. Taken together, these two studies provide additional information regarding the rare manifestation of AMP and its genetic influence on AS patients.

Several case reports examined unusual ocular symptoms that were present in patients with AS. Stanojcic et al. [46] published a case report regarding an AS patient who was a middle-aged Caucasian male and had an unusual triad of features: ‘stair-case' fovea, choroidal thinning, and peripheral retinoschisis. ‘Stair-case' fovea is characterized by vitreomacular traction and is a possible precursor to macular holes. This condition is linked to abnormalities in type IV collagen. Choroidal thinning may cause reduced blood flow and vision. Finally, peripheral retinoschisis is a condition in which the layers of the retina separate, causing vision loss. Along with unusual symptoms, however, this patient also presented with multiple common ocular abnormalities, such as lenticonus, cataracts, macular holes, and perimacular retinopathy. No treatment was reported in this case report. Ghadiri et al. [35] reported a case in a middle-aged Caucasian male patient who presented with unusual symptoms in his left eye that was described as the same triad of features reported in Stanojcic et al.: “stair-case” fovea, choroidal thinning, and peripheral retinoschisis. These signs were associated with unique mutations in *COL4A5*. The patient also presented with macular holes, and a vitrectomy was performed to repair the holes. No further treatment was offered due to the risk of instability after the vitrectomy. Ghadiri et al. suggest that these features can be useful in the diagnosis and prediction of additional ocular abnormalities, the timing of renal failure, and even the mode of inheritance for AS. Igami et al. [39] examined a case report with a rare ocular diagnosis. The middle-aged Caucasian AS female patient arrived at the clinic and was found to have bilateral macular atrophy. Although the patient also had lenticonus and macular thinning, macular atrophy was a rare finding that has never been directly linked to AS. Igami et al. end the report by emphasizing that macular atrophy is an important, rare abnormality that needs to be recognized by ophthalmologists to ensure an accurate diagnosis of AS. Although no current treatment was reported in this case report, the author briefly mentioned that gene therapy could have the potential to effectively treat this disorder. Finally, a case report by Yasuzumi et al. [50] discusses a teenage patient with AS who was experiencing chronic renal failure and developed bilateral retinal detachment. Yasuzumi et al. investigated whether renal failure can lead to unusual conditions, such as retinal detachment. The physicians in the case decided to target renal failure by utilizing intensive peritoneal hemodialysis. Surprisingly, the retinal detachment was resolved, and vision recovered following peritoneal hemodialysis treatment. Since high blood pressure can weaken capillaries and cause the retina to detach, peritoneal dialysis would lower the blood pressure thus resolving the retinal detachment.

Moshirfar et al. [43] detailed a female AS patient who presented with keratoconus, a rare AS condition where the cornea pushes outward and develops into a cone shape. Keratoconus is a type of corneal ectasia and could potentially occur in AS patients due to genetic factors such as a mutation in the PAX6 gene. Although the presentation of keratoconus with AS is rare, Moshirfar et al. recommend further studies to examine if there is an underlying connection between the two diseases.

The presentation of vitelliform lesions in AS patients is another rare retinal abnormality. In the study by Thomas et al. [47], they found that one of the two AS patients examined presented with a vitelliform lesion of the macula, a genetically inherited abnormality that can cause loss of central vision. This patient was only the second patient reported to have a vitelliform lesion. Due to the identification of photoreceptor peripherin gene mutations, the researchers hypothesized that mutations in the collagen gene in AS could lead to the formation of vitelliform lesions. Furthermore, a case report by Eriksen and Jørstad [30] described an AS patient who presented with multiple vitelliform lesions (MVL). The authors of the report emphasized that this MVL finding is rare and was not known to be reported by previous studies of AS. Although the researchers acknowledged that the pathophysiology of multiple vitelliform lesions in the AS patient is unclear, it is hypothesized that the type IV collagen affected in AS can lead to an unstable Bruch's membrane (BM). If the strength of the BM is ultimately compromised, metabolic by-product levels would increase and multiple vitelliform lesions would appear.

### 3.4. Potential Medical Treatment of Ocular Abnormalities

AS ocular effects have yet to be proven to be alleviated by surgical approaches, such as vitrectomies, cataract surgeries, and IM peeling [29, 32, 35, 37, 38, 47]. These techniques have been used to help target ocular findings and complications in non-AS patients. Although some papers mentioned these surgical techniques in a general patient population, it must be noted that these surgical approaches for AS patients have not been shown to be effective in a large study. For example, one study explained that ophthalmologists should be cautious when considering a vitrectomy in AS patients due to a reduced retinal scaffold [35]. Vitrectomies are used to repair macular holes, which is a break in the macular region of the retina [51]. A vitrectomy is performed in two parts. First, any vitreous fluid on the macula and the hole is removed. Second, a temporary bubble of gas is placed into the eye. Then, the eye will heal as the gas bubble holds the macula in place. In rare circumstances, a proposed treatment option is surgical internal limiting membrane (ILM) peeling which includes removal of the basal lamina of the inner retina. In this procedure, the membranes around the edges of the hole that can promote macular hole formation are removed. Although this procedure generally has a high success rate, there are complications that can arise in AS patients with weakened retinal stability leading to vision loss [40]. One study referenced cataract surgery as a potential solution to vision loss following lenticonus but noted how the procedure can be difficult in certain patients due to elasticity of the thin capsular bag during the capsulorrhexis [29]. Although cataract surgeries are common, patients with AS seem to develop cataracts at higher rates due an increase of small ruptures in the lens capsule [28, 29, 41, 42, 44, 45]. A cataract is diagnosed by a visual acuity test or slit-lamp examination. Cataract surgery is performed by removing the lens and replacing it with an artificial lens.

Another option, which will continue to be explored, is gene therapy [39]. Gene therapy is a technique that either alters or replaces the abnormal gene with the correct form of the gene. As previously mentioned, it must be noted that gene therapy is not able to treat fetal and early developmental alterations of AS. In theory, researchers are still studying how introducing a normal copy of the collagen gene, *COL4A3*, *COL4A4*, or *COL4A5*, could potentially allow the human body to produce collagen near the eye. Thus, the ocular symptoms could be alleviated as collagen plays a role in the healing processes, function, and structure of the eye. With the growing field of gene therapy and its potential to treat rare genetic disorders, it could become a potential treatment option for patients in the coming decades. However, currently, gene therapy is not a proven treatment option for genetic diseases, but initial results in treating both AS podocyte-lineage cell lines and COL4A5 truncating variants have shown promise [20, 52].

## 4. Discussion

### 4.1. Main Findings and Implications

Knowledge of AS and other genetic disorders has the potential to alter how medical professionals examine their patients and pursue medical management strategies. In this review, multiple reports summarized the ocular abnormalities presented in Alport syndrome and potential treatments. This systematic review reports that lenticonus, retinopathy, macular holes, retinal thinning, macular thinning, cataract, and dull macular reflex are extremely common in AS patients presenting with ocular abnormalities. Several physicians and researchers have reported associations between defects in the retina and lens with AS (see Table 1). This is due to a lack of collagen production in the visual area leading to a deterioration of vision or other eye anomalies. Furthermore, severe vision complications, such as blindness, retinal detachment, and vitelliform lesions can occur over time in patients. We found no definitive conclusion regarding the validity, safety, or efficacy of treatment options such as surgery and gene therapy for AS patients. For example, although non-AS patients may recover well following vitrectomy for macular hole repair, this is not the case for AS patients who have a reduced retinal scaffold that can cause a poor postoperation prognosis [45]. The risk that ophthalmologists must keep in mind is that AS patients may respond negatively and develop permanent blindness following any surgical intervention. There were no studies in this review that specifically investigated the impact of surgical intervention or gene therapy on ocular complications in AS patients. Thus, further research into AS treatments is warranted and ongoing in the scientific field.

The findings of a high occurrence of certain ocular irregularities among patients of different backgrounds are encouraging. The papers that were included in the review were diverse in regard to patients, design, follow-up, and data extraction. Although we remain cautious in making definitive conclusions, there is a consistency in the types of health complications that present in AS patients. Although the disorder is rare, the findings in this review reinforce that training in the recognition of symptoms of AS is necessary for patient care. In addition, the family history of the patient should be examined for possible genetic inheritance patterns. This knowledge can aid in the early diagnosis and management of AS through other options such as medications. Many researchers have mentioned a strong association between an ophthalmologist performing a thorough ophthalmic examination and confirming an AS diagnosis. As genetic testing may not always be accessible, an ophthalmic examination with an OCT, slit-lamp examination, and retinal photography can be effective for patients at risk. Unfortunately, many physicians may overlook certain AS ocular complications as they may be subtle and not severely impact the vision of the patients. Utilizing the knowledge of AS and its respective complications is crucial in the field of medicine as ophthalmologists can potentially aid other physicians in verifying an AS diagnosis.

### 4.2. Limitations

Although the broad range of symptoms among patients from various demographics enhances this review's generalizability and the study is conducted in accordance with PRISMA guidelines, there are limitations of the evidence and review processes that must be considered. The study selection process only included English papers, so there may have been other relevant papers in other languages that were excluded. Due to the rarity of the disorder in the general population, there is a small sample size of patients and the total number of cases. Third, the identified reports on AS may only represent a fraction of the actual cases due to many patients who have AS but were misdiagnosed with another disorder with similar health complications. Another limitation of this review was the fact that many papers did not explore long-term check-ups with the patients. This is an issue as many AS patients could have developed new or worsened ocular complications over time. Finally, as the majority of the papers did not discuss potential medical treatment options, we did not have sufficient information to make a conclusion about the future effectiveness of potential treatments such as surgical techniques or gene therapy. These limitations were diminished by the following: (1) utilizing strong keywords and criteria to identify relevant research papers and (2) investigating any source of heterogeneity, even though this was not significant for our review. Since symptoms were all recorded during physician visits in published articles, no recall bias or response bias was present within any study part of the review.

## 5. Conclusion

Lenticonus, retinopathy, macular holes, retinal thinning, macular thinning, cataract, and dull macular reflex were present in the majority of AS patients. At least one of the preceding findings was present in 99.4% of the studied AS patients. The rarer complications of choroidal thinning, retinal detachment, keratoconus, and vitelliform lesions were only reported in about 3.3% of the AS patients that were studied. It is crucial that ophthalmologists are aware of these complications to identify them, aid in the diagnosis of the patient, and provide the proper treatment. A practical method would be to track the patient's family history to identify common trends related to AS in previous generations, such as renal disease, auditory degeneration, and ocular abnormalities. The reviewed literature mentions no standardized medical intervention for AS ocular abnormalities. Surgical techniques, such as vitrectomies, and gene therapy have not been evidenced in a large research study to manage the disorder. Thus, physicians must proceed carefully when evaluating potential interventions for AS patients who are more susceptible to further complications after the operation.

Further research within patient populations worldwide would support a greater understanding of AS and the treatments currently utilized in other countries. Exposure and information about this disorder can encourage patients, physicians, and researchers to support family history tracking, preventative strategies, and treatment options for AS.

## Figures and Tables

**Table 1:** Summary of ocular abnormalities in AS patients from 1977 to 2022.

Authors	Sample size (individuals)	Ocular abnormalities	Potential treatment	Sample size (eyes)
Adıyeke et al. [28]	21	LE, RE, MT, C, MH	N/A	N/A
Ahmed et al. [29]	32	LE, RE, MT, RT, C, MH	Cataract surgery	63
Eriksen and Jørstad [30]	1	LE, MT, MVL	N/A	2
Fawzi et al. [31]	9	LE, RE, RT, MH, VL, RD	N/A	18
Fukukita et al. [32]	26	LE, RE, MT, MH	N/A	26
Gehrs et al. [33]	1	MF, RE	N/A	1
Gelisken et al. [34]	13	RE, RVT	N/A	N/A
Ghadiri et al. [35]	1	SFS, LE, CT, LPS, MH	N/A	2
Govan [36]	16	LE, PFRE, MF, DMR	N/A	32
Gupta et al. [37]	2	LE, MT, PFRE	N/A	4
Hess et al. [38]	14	LE, RE, DMR	N/A	28
Igami et al. [39]	1	LE, MT, PFRE	Gene therapy	N/A
Jeffrey et al. [40]	20	LE, RE, PF	N/A	N/A
Karki and Shrestha [41]	1	LE, MF, C	N/A	2
Mete et al. [42]	8	LE, MF, C, ALCA, MH	N/A	16
Moshirfar et al. [43]	1	LE, RE, PFRE, KE	N/A	2
Nielsen [44]	6	LE, DMR, C, AMP	N/A	12
Singh et al. [45]	4	LE, RD, C, AMP	N/A	8
Stanojcic et al. [46]	1	SFS, DMR, RE, PFRE, C	N/A	1
Thomas et al. [47]	2	MH, RE, RT, MT, VL	N/A	4
Wong et al. [48]	1	LE, RE, RT, MT	N/A	2
Xu et al. [49]	13	LE, MF, C, NY	N/A	N/A
Yasuzumi et al. [50]	1	RD	Peritoneal dialysis	2

LE, lenticonus; MF, macular flecks; RE, retinopathy; PFRE, perimacular fleck retinopathy; MT, macular thinning; MH, macular hole; CT, choroidal thinning; RT, retinal thinning; LPS, late-stage peripheral schisis; C, cataract; RD, retinal detachment; DMR, dull macular reflex; AMP, abnormal macular pigmentation; VL, vitelliform lesion; MVL, multiple vitelliform lesions; SFS, “stair-case” foveal sign; KE, keratoconus; NY, nystagmus; RVT, retinal vascular tortuosity; ALCA, anterior lens capsule abnormalities; N/A, not applicable.

## Abbreviations

AS: Alport syndrome
OTC: Optical coherence tomography
LOVD: Leiden open variation database
CASP: Critical appraisal skills program
RCTs: Randomized controlled trials
FAZ: Foveal avascular zone
FAF: Fundus autofluorescence
LE: Lenticonus
MF: Macular flecks
KE: Keratoconus
RE: Retinopathy
PFRE: Perimacular fleck retinopathy
MT: Macular thinning
MH: Macular hole
CT: Choroidal thinning
RT: Retinal thinning
LPS: Late-stage peripheral schisis
RD: Retinal detachment
DMR: Dull macular reflex
AMP: Abnormal macular pigmentation
VL: Vitelliform lesion
MVL: Multiple vitelliform lesions
SFS: “Stair-case” foveal sign
ILM: Internal limiting membrane
ALC: Anterior lens capsule
ALCA: Anterior lens capsule abnormalities
NY: Nystagmus
RVT: Retinal vascular tortuosity.

## Additional Points

*Strengths and Limitations of This Study*. (i) This review summarizes the common and rare ocular abnormalities found in AS patients along with the medical management and treatments that are utilized for each type of abnormality. (ii) The identified reports in this review on AS have small sample sizes and may only represent a fraction of the actual cases due to many patients who have AS but were diagnosed with another disorder. (iii) Studies identified in this review have a limited discussion on potential medical treatment options, thereby restricting a discussion on the future effectiveness of potential treatments such as surgical techniques or gene therapy.
